# Inflammation-Associated Nitrotyrosination Affects TCR Recognition through Reduced Stability and Alteration of the Molecular Surface of the MHC Complex

**DOI:** 10.1371/journal.pone.0032805

**Published:** 2012-03-14

**Authors:** Chaithanya Madhurantakam, Adil D. Duru, Tatyana Sandalova, John R. Webb, Adnane Achour

**Affiliations:** 1 Centre for Infectious Medicine, Department of Medicine Huddinge, Karolinska University Hospital Huddinge, Karolinska Institutet, Stockholm, Sweden; 2 Trev and Joyce Deeley Research Centre, British Columbia Cancer Agency, Victoria, British Columbia, Canada; Saint Louis University School of Medicine, United States of America

## Abstract

Nitrotyrosination of proteins, a hallmark of inflammation, may result in the production of MHC-restricted neoantigens that can be recognized by T cells and bypass the constraints of immunological self-tolerance. Here we biochemically and structurally assessed how nitrotyrosination of the lymphocytic choriomeningitis virus (LCMV)-associated immunodominant MHC class I-restricted epitopes gp33 and gp34 alters T cell recognition in the context of both H-2D^b^ and H-2K^b^. Comparative analysis of the crystal structures of H-2K^b^/gp34 and H-2K^b^/NY-gp34 demonstrated that nitrotyrosination of p3Y in gp34 abrogates a hydrogen bond interaction formed with the H-2K^b^ residue E152. As a consequence the conformation of the TCR-interacting E152 was profoundly altered in H-2K^b^/NY-gp34 when compared to H-2K^b^/gp34, thereby modifying the surface of the nitrotyrosinated MHC complex. Furthermore, nitrotyrosination of gp34 resulted in structural over-packing, straining the overall conformation and considerably reducing the stability of the H-2K^b^/NY-gp34 MHC complex when compared to H-2K^b^/gp34. Our structural analysis also indicates that nitrotyrosination of the main TCR-interacting residue p4Y in gp33 abrogates recognition of H-2D^b^/gp33-NY complexes by H-2D^b^/gp33-specific T cells through sterical hindrance. In conclusion, this study provides the first structural and biochemical evidence for how MHC class I-restricted nitrotyrosinated neoantigens may enable viral escape and break immune tolerance.

## Introduction

The biological function of proteins and cellular metabolism can be significantly modulated through post-translational modifications (PTMs) [1], [2]. Naturally occurring PTMs can either result from enzyme modifications or arise spontaneously [3], [4]. However, PTMs can also be used by bacterial and viral pathogens to compromise critical immune responses and host factors acting against infection [5]. The breakdown of immunological tolerance resulting from such modifications may render endogenous antigens immunogenic [3], [6]. A large array of PTM peptides presented by major histocompatibility complex (MHC) class I and class II molecules have now been described [7], [8], and it has been demonstrated that modifications such as cysteinylation [9], [10], glycosylation [11], [12] or phosphorylation [13], [14] may affect T cell immunoreactivity, resulting in immune escape and/or initiation of autoimmunity.

Protein tyrosine nitration is a hallmark of inflammation, and is associated with up-regulated expression of inducible nitric oxide synthase (iNOS) [15], [16]. Nitrotyrosinated proteins (NT-proteins) are generated *in vivo* by nitration with peroxynitrite, itself derived from nitric oxide and superoxide which are both released from activated inflammatory cells [17]. NT-proteins may accumulate in apoptotic or inflamed tissues [18]. Indeed, accumulation of the amino acid homolog 3′-nitrotyrosine (NY) and of NT-proteins has been observed at inflammatory sites in Alzheimer's disease [19], arthritis [20], [21], [22], [23], atherosclerosis [22], [23], autoimmune diabetes [24], autoimmune uveitis [25], celiac disease [26], ischemia-reperfusion injury [27], multiple sclerosis [28], Parkinson's disease [29], respiratory disease [30] and transplant rejection [31], as well as in various cancers and infectious diseases [32]. NT self-proteins may thus be highly immunogenic, eliciting both humoral and cellular responses [17], [33].

The potential immunogenicity of MHC class II-restricted NT autologous peptides has been previously investigated using the I-E^k^-restricted T cell pigeon/moth cytochrome c (PCC/MCC_88–103_) [34] as well as the I-A^k^-restricted hen egg-white lysozyme (HEL) epitopes [35]. Both studies demonstrated unequivocally that conversion of tyrosine to nitrotyrosine resulted in dramatic consequences for T cell recognition. Indeed, processing of native proteins by activated antigen presenting cells (APCs) resulted in the presentation of MHC class II-restricted NT-epitopes in lymphoid tissues, significantly altering specific T cell responses and eliciting NT-specific CD4^+^ T cells that evaded negative selection in the thymus and thus central tolerance [33]. Likewise loss of tolerance by NT-specific CD4^+^ T cells has recently been shown to be critical for the production of autoreactive antibodies [36]. These studies indicate that NT-proteins generated during inflammation might constitute an important class of neoantigens that could promote autoimmune T cell responses.

The well-established lymphocytic choriomeningitis virus (LCMV) glycoprotein system was also used by Hardy *et al* to demonstrate that conversion of tyrosine to NY also profoundly affected T cell recognition of MHC class I-restricted epitopes [37]. A significant amount of the overall CD8^+^ T cell response to LCMV is dominated by very few viral epitopes, comprising the H-2D^b^-and H-2K^b^- restricted peptide gp33–41 (hereafter referred to as gp33; KAVYNFATC) and the H-2K^b^-restricted peptide gp34–41 (gp34; AVYNFATC) [38], [39]. Both gp33 and gp34 contain a single tyrosine residue at positions 4 (p4Y) and 3 (p3Y), respectively. T cell populations, exclusively specific to the nitrotyrosinated MHC complexes H-2D^b^/NY-gp33, H-2K^b^/NY-gp33 and/or H-2K^b^/NY-gp34 [37], were elicited in C57/BL6 mice (H-2D^b+^/H-2K^b+^) following immunization with the nitrated peptide NY-gp33. Importantly, CD8^+^ T cell hybridomas specific for NY-gp33 comprised two distinct subsets recognizing either H-2D^b^/NY-gp33 or H-2K^b^/NY-gp33. While the T cell hybridoma 24H1 responded to stimulation with both H-2K^b^/NY-gp34 andH-2K^b^/NY-gp33, it did not recognize the unmodified wild-type MHC complex H-2K^b^/gp34 nor H-2K^b^/gp33 [37]. Similarly, the H-2D^b^/NY-gp33-specific T cell hybridoma 4C8 did not recognize H-2D^b^/gp33. In contrast, nitrotyrosination of the main T cell receptor (TCR)-interacting peptide tyrosine residue p4Y abrogated recognition of H-2D^b^/NY-gp33 MHC complexes by H-2D^b^/gp33-specific T cells from P14 transgenic mice [37].

The present study provides a comparative biochemical and structural analysis that explains how nitrotyrosination of the LCMV-associated immunodominant epitopes gp33 and gp34 can alter T cell recognition in the context of the two different MHC class I molecules H-2D^b^ and H-2K^b^. Nitrotyrosination of the MHC-restricted peptide impairs TCR recognition through reduced stability and alteration of the molecular surface of the MHC complex. The possible implications for the role of nitrotyrosination in the creation of modified neoantigens that allow for viral escape and/or breaking of immune tolerance that possibly results in autoimmune disorders are discussed.

## Materials and Methods

### Preparation and crystallization of H-2K^b^in complex with gp34 and NY-gp34

Peptides gp34 (AVYNFATM) and NY-gp34 (AV-p3NY-NFATM) were purchased from Genscript (Piscataway, NJ, USA). Refolding and purification of MHC/peptide complexes were followed according to previously published protocols [40], [41], [42], [43]. The best crystals for H-2K^b^/gp34 and H-2K^b^/NY-gp34 were obtained in hanging drops by vapor diffusion in 2.1 M NaH_2_PO_4_/K_2_HPO_4_ (pH 6.4), 1.5% MPD and 1.8 M NaH_2_PO_4_/K_2_HPO_4_ (pH 6.7), 1.5% MPD, respectively. Typically, 2 µl of 6 mg/ml protein were equilibrated against 2 µl of crystallization reservoir solution at 20°C.

### Data collection and processing

Data collection was performed under cryogenic conditions (temperature 100 K) at beam lines ID14-2 and ID14-4 at the synchrotron radiation facility at ESRF (Grenoble, France) to a resolution of 2.0 and 2.6 Å for H-2K^b^/gp34 and H-2K^b^/NY-gp34, respectively. Crystals were soaked in a cryoprotectant solution containing 25% glycerol before data collection. A total of 180 and 360 images were collected with 0.5° oscillation per frame for both H-2K^b^/gp34 and H-2K^b^/NY-gp34. Data were processed with MOSFLM [44] and SCALA from the CCP4 suite [45]. The space group was determined to be *P2_1_2_1_2_1_* for H-2K^b^/gp34 with unit-cell parameters *a* = 88.4, *b* = 92.6, *c* = 128.8 Å. The space group for H-2K^b^/NY-gp34 was*P2_1_* with unit-cell parameters *a* = 50.5, *b* = 88.5, *c* = 119 Å, *α* = *γ* = 90.0° and *β* = 94.7°. Data collection statistics are provided in Table 1.

**Table 1:** Data Collection and Refinement Statistics.

	H-2K^b^-gp34	H-2K^b^-NYgp34
**PDB code**	3ROO	3ROL
**Cell parameters (Å)**	a = 88.4, b = 92.6, c = 128.8, α = β = γ = 90.0	a = 50.5, b = 88.5, c = 119.0, α = γ = 90.0, β = 94.7
**Data Collection**		
**Space group**	*P 2_1_ 2_1_ 2_1_*	*P 1 2_1_ 2*
**Resolution range (Å)**	57.3 – 2.0	50.3 – 2.6
**Number of molecules/asymmetric unit**	2	2
**Number of reflections**		
**Observed**	338476 (47869)	138558 (20164)
**Unique**	72101 (10411)	32286 (4682)
***I/σ (I)***	11.6 (2.6)	12.9 (3.3)
**Completeness (%)**	99.9 (100.0)	100.0 (100.0)
1 ***R_sym_ (%)***	8.3 (67.0)	11.9 (39.8)
**Multiplicity**	4.7 (4.6)	4.3 (4.3)
**Refinement Statistics**		
2 ***R_cryst_*** **(%)**	22.5	24.7
3 ***R_free_*** ** (%)**	26.7	29.5
**Number of protein atoms**	6695	6391
**Water/other molecules**	396/4	283/4
**rmsd from ideal geometry**		
**Bond length (Å)**	0.009	0.010
**Bond angle (°)**	1.136	1.285
**Ramachandran Plot (%)**		
**Residues in preferred regions**	96.25	95.95
**Residues in allowed regions**	3.75	4.05
**Non-glycine residues in disallowed regions**	0	0

Values in parentheses are for the highest resolution shell.

1 *R_sym_* = Σ*_h_*Σ*_i_|I_h,i_−I_h_|*/Σ*_h_*Σ*_i_I_h,i_*, where *I_h_* is the mean intensity of the *i* observations of symmetry related reflections of *h*.

2 *R_cryst_* = Σ|*F_obs_*−*F_calc_*|/Σ*F_obs_*, where *F*_obs_ and *F*_calc_ are the observed and the calculated structural factors, respectively.

3 *R*_free_ was calculated using 5% of the reflections.

### Crystal structure determination and refinement

The crystal structure of H-2K^b^/gp34 was solved by molecular replacement (MR) using Phaser [46] and the H-2K^b^/GNYSFYAL MHC complex (PDB code 1LK2) as a search model. Five percent of the total reflections were set aside for monitoring refinement by *R_free_*. The crystal structure of H-2K^b^/NY-gp34 was solved thereafter by MR using H-2K^b^/gp34 as a search model. Refinement of the two crystal structures was performed using REFMAC5 [47]. After each round of refinement, missing residues were added in successive cycles of manual building followed by restrained refinement cycles in REFMAC5 [47]. The final refinement parameters are presented in Table 1. Figures were created using the program PyMOL [48].

### Peptide-MHC binding affinity assays

Peptide-MHC binding affinity assays were performed using transporter associated with antigen processing (TAP)-deficient RMA-S cells as described previously [49], by assessing the capacity of the different peptides to stabilize cell surface expression of H-2K^b^ complexes. Briefly, 5×10^5^ RMA-S cells were pulsed with different concentrations of indicated peptides in serum free AIM-V medium (Invitrogen, Carlsbad, CA, USA) at 26°C overnight in 5% CO_2_. Cells were subsequently washed and incubated in AIM-V medium at 37°C for 60 min in the absence of peptides. Cells were then washed twice with PBS before staining with anti-H-2K^b^ AF6-88.5 (BD PharMingen/BD Biosciences, MountainView, CA, USA). Following washing and fixation in 1% PFA, H-2K^b^ cell surface expression was detected by flow cytometry on BD FACSCalibur (BD Biosciences). Flow cytometry data was analyzed using Cell Quest Pro (BD Biosciences). The mean fluorescence intensity (MFI) of H-2K^b^ expression for the indicated peptide concentrations was divided by the observed MFI on cells without peptide as an estimate of peptide binding. The HIV-derived H-2D^d^-restricted epitope P18 (RGPGRAFVTI) was used as a negative control while the H-2K^b^-restricted Moloney murine leukemia virus peptide MULV (SSWDFITV) was used as a positive control.

### Assessment of MHC complex stability using circular dichroism (CD) analysis

CD measurements were performed in 20 mM K_2_HPO_4_/KH_2_PO_4_ (pH 7.5) using protein concentrations from 0.15 to 0.25 mg/ml. Spectra were recorded with a JASCO J-810 spectropolarimeter (JASCO Analytical Instruments, Great Dunmow, UK) equipped with a thermoelectric temperature controller in a 2 mm cell. pMHC denaturation was measured between 30°C and 75°C at 218 nm with a gradient of 48°C/hour at 0.1°C increments and an averaging time of 8 seconds. The melting curves were scaled from 0% to 100% and the T_m_ values extracted as the temperature at 50% denaturation. Curves and T_m_-values are an average of at least two measurements from two independent refolding assays per MHC complex. Spectra were analyzed using GraphPad Prism 5 and T_m_ values compared using an unpaired, two-tailored T test.

### Assessment of MHC complex stability using Thermofluor analysis

Differential scanning fluorimetry experiments were carried out using an iQ5 real-time PCR detection system (Bio-Rad). 96-well PCR plates were filled with 23 µl of MHC solution (0.25 mg/mL) in 10 mM Na^+^ HEPES buffer (pH 7.3), 150 mM NaCl per well. Finally, the fluorophore (2 µl of 125× SYPRO Orange (Sigma S5692)) was added to each well and the plate was sealed with optical sealing tape (Bio-Rad). An iQ5 Real-Time PCR Detection System, calibrated with External Well factor solution was used to monitor the changes in fluorescence intensity of the fluorophore. The temperature of the samples was changed from 20 to 95°C at a heating rate of 1°C/min and the fluorescence was recorded every 0.2°C. The melting temperature (T_m_) of each melting curve was calculated as the maximum of the first derivative of the curve. The average T_m_ value of each protein was determined from three distinct melting curves.

### Molecular modeling of TCR in complex with wild-type and nitro-tyrosinated MHC complexes

Molecular models of the TCR P14 in complex with H-2D^b^/gp33 or H-2D^b^/NY-gp33 were created using previously described protocols [50], [51]. Interactions between P14 and each MHC complex were analyzed visually for improper sterical contacts. Docking of P14 on its MHC/peptide ligand was also assessed according to contacts between evolutionary conserved amino acids suggested to be important for TCR/MHC interaction [52]. The molecular model was subjected to several rounds of energy minimization using the CNS suite of programs [53]. The coordinates of the TCR/MHC/peptide complexes will be provided upon request.

## Results and Discussion

### Overall structures of H-2K^b^ in complex with wild-type gp34 and nitrotyrosinated NY-gp34

**Figure 1:**
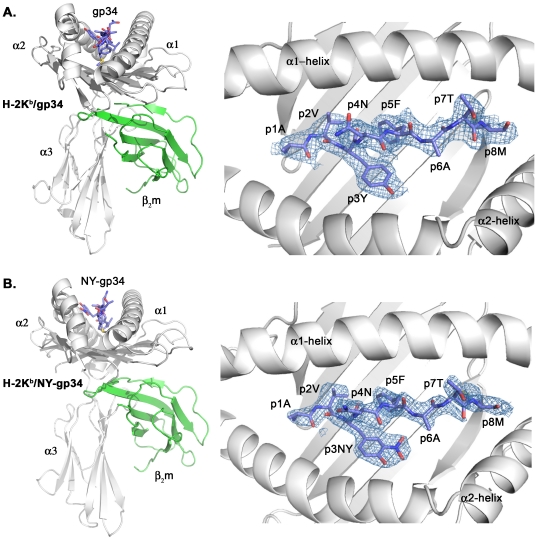
Crystal structures of H-2K^b^ in complex with gp34 and NY-gp34. Overall schematic views of the H-2K^b^/gp34 and H-2K^b^/NY-gp34 MHC complexes are presented in the left part of panels A and B. The α1, α2 and α3 domains of the MHC heavy chain are colored in white. The β_2_m subunit is colored in green. The peptides are in blue. The 2F_o_-F_c_ electron density maps for the peptides gp34 and NY-gp34 when bound to H-2K^b^ presented in the right part of panels A and B, respectively, are contoured at 1.0 σ. The final models are displayed for comparison. The peptides, depicted with their N-termini to the left and their C-termini to the right, are displayed ‘from above’ as seen by the TCRs.

The three dimensional structures of H-2K^b^ in complex with the wild-type gp34 (AVYNFATM) and with the nitrotyrosinated NY-gp34 (AV-p3NY-NFATM) were determined to 2.0 and 2.6 Å resolutions, respectively (Table 1). Both crystal structures displayed good stereochemistry. The final electron density maps are of good quality with well-defined polypeptide chains (Figures 1A and 1B). In particular, the electron densities for all H-2K^b^ residues in contact with the peptides gp34 and NY-gp34 are clearly defined. The overall three-dimensional structures of H-2K^b^/gp34 and H-2K^b^/NY-gp34 are remarkably similar with a root mean square deviation (rmsd) of 0.37 Å^2^ for the entire MHC complexes (heavy chains in complex with peptides and β_2_-microglobulin (β_2_m)) and of 0.27 Å^2^ for heavy chain residues 1–176 corresponding to the α_1_ and α_2_ domains.

### Protrusion of residue p1K in H-2K^b^/gp33 does not alter the conformation of MHC residues within the N-terminal part of the peptide-binding cleft

**Figure 2:**
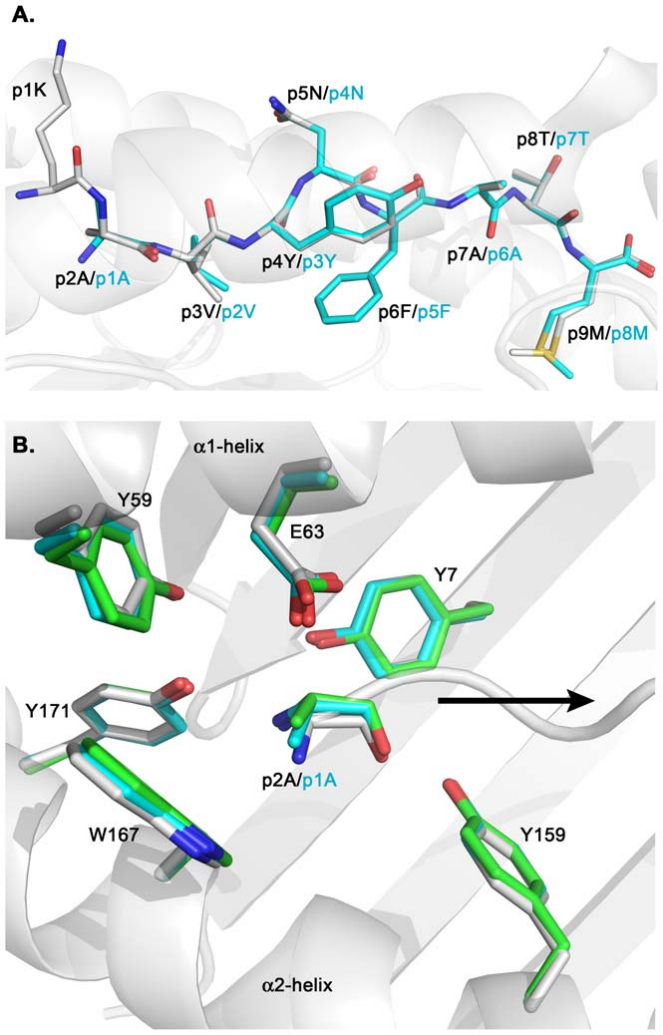
Protrusion of p1K in H-2K^b^/gp33 does not affect the overall conformation of the N-terminal part of the peptide binding cleft and conserves the conformation of the epitopes. **A.** Superimposed side views of the peptides gp33 (KAVYNFATM) and gp34 (AVYNFATM) depicting how residue p1K in gp33 protrudes out of the peptide binding cleft of H-2K^b^. The remaining residues of gp33 take a similar conformation to all the side chains of gp34. The peptides gp33 and gp34, annotated in black and cyan, respectively, are depicted with their N termini to the left and their C termini to the right. The carbon atoms of the peptides gp33 and gp34 are colored in white and cyan, respectively. Carbon, nitrogen and oxygen atoms are in cyan, blue and red, respectively. The peptide-binding cleft of H-2K^b^ is colored white. **B.** Conformation of side chain residues interacting with the N-termini of peptides in the crystal structures of H-2K^b^/gp33 (in white) and H-2K^b^/gp34 (both MHC complexes from the asymmetric unit are displayed in cyan and light green, respectively), following superposition of the α_1_α_2_ domains. Note that the p2A residue in gp33 occupies the position corresponding to the p1A in gp34. The side chain of p1K in gp33 is not displayed. The orientation of the peptides is depicted by a black arrow (from the N terminus toward the C terminus). The α1 and α2 helices are indicated.

The previously determined crystal structure of H-2K^b^ in complex with gp33 (KAVYNFATM) revealed that the nonameric peptide binds to H-2K^b^ as an octamer with the stretch of residues AVYNFATM filling the peptide-binding cleft, while the highly flexible residue p1K extended out of the binding groove [50] (Figure 2A). As for p2A in H-2K^b^/gp33, the first peptide residue p1A in both H-2K^b^/gp34 (AVYNFATM) and H-2K^b^/NY-gp34 (AV-p3NY-NFATM) is surrounded by a cluster of tyrosine residues (Y7, Y159 and Y171) that forms a network of hydrogen bond interactions with the nitrogen atom of p1A. However, although the protrusion of p1K out of the peptide-binding cleft in H-2K^b^/gp33 results in a significant modification of the position of the nitrogen atom of residue p2A when compared to p1A in H-2K^b^/gp34 or H-2K^b^/NY-gp34, this does not affect the conformation of the cluster of tyrosine residues that surround the N-terminal part of the peptide (Figure 2B). Furthermore, the conformation of the stretch of peptide residues AVYNFATM bound within the peptide-binding cleft is very similar in both H-2K^b^/gp33 and H-2K^b^/gp34 (Figure 2A). Thus although the N- and C- peptide termini play crucial roles in binding of epitopes within the peptide-binding cleft formed by the α_1_α_2_ groove [54], these are not always necessarily required. It has been previously demonstrated that longer peptides may protrude out of the groove and that modified and/or shorter peptides can still bind efficiently within the groove through the use of a network of water molecules within the peptide binding cleft [50], [55], [56], [57].

In the prior study by Hardy *et al*, T cells could be readily elicited in C57/BL6 mice following immunization with NY-gp33 [37]. Interestingly, while the H-2K^b^/NY-gp33-specific CD8^+^ T cell hybridoma 24H1 recognized H-2K^b+^ target cells coated with both NY-gp33 and NY-gp34, it did not recognize H-2K^b^/gp34 MHC complexes. Furthermore, it should also be noted that 24H1 T cells recognized H-2K^b^/NY-gp34 to a higher level when compared to H-2K^b^/NY-gp33. The relatively lower recognition of H-2K^b^/NY-gp33 by 24H1 could be explained by the protrusion of the side chain of p1K from the N-terminal part of the peptide-binding cleft of H-2K^b^/NY-gp33, which could potentially limit optimal recognition by the TCR. However, the question remained as to why the H-2K^b^/NY-gp33 and H-2K^b^/NY-gp34-specific TCR of 24H1 did not recognize H-2K^b^/gp34?

### Nitrotyrosination of peptide residue p3Y induces a profound conformational change of the H-2K^b^ residue E152, altering TCR recognition

**Figure 3:**
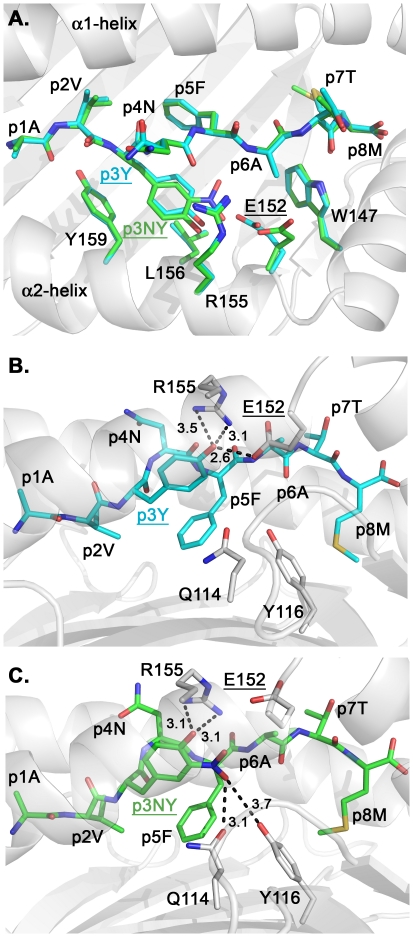
Nitrotyrosination of peptide residue p3Y results in a conformational change of the side-chain of the H-2K^b^ residue E152 only, altering TCR recognition. **A.** Superposition of the peptide-binding clefts of H-2K^b^/gp34 and H-2K^b^/NY-gp34 demonstrate a subtle shift of the side chain of p3NY when compared to p3Y, resulting in a significant conformational change of the TCR-interacting H-2K^b^ heavy chain residue E152 (underlined). Both side chains of residues p3Y and p3NY protrude in the D-pocket of H-2K^b^ consisting of residues W147, E152, R155, L156 and Y159. The gp34 and NY-gp34 peptides are colored cyan and light green, respectively. **B.** Side view of the peptide gp34 when bound to H-2K^b^. Three hydrogen bond interactions are formed between p3Y and the H-2K^b^ residues E152 and R155. No interactions are observed with the H-2K^b^ residues Q114 and Y116. **C.** A novel hydrogen bond and a long ionic range interaction are formed between p3NY and the H-2K^b^ residues Q114 and Y116, respectively. While two hydrogen bond interactions are maintained between p3NY and R155, all interactions are lost with E152.

The conformations of the peptides gp34 and NY-gp34 are very similar when bound to H-2K^b^, besides a lateral shift of 0.7 Å of the side chain of the nitrotyrosine p3NY in H-2K^b^/NY-gp34 when compared to the conformation of p3Y in H-2K^b^/gp34 (Figure 3A). In H-2K^b^/gp34 the side-chain of p3Y projects down into the D-pocket formed by residues Q114, E152, R155, L156 and Y159 (Figures 3A and 3B). Three hydrogen bond interactions are formed between the side chain of p3Y and the side chains of the H-2K^b^ residues E152 and R155 (Figure 3B). Nitrotyrosination of p3Y alters only the conformation of the H-2K^b^ heavy chain residue E152 which, in order to accommodate the side-chain of p3NY, undergoes a large conformational modification in H-2K^b^/NY-gp34 when compared to H-2K^b^/gp34 (Figure 3C). In contrast, the rest of the H-2K^b^ peptide-binding groove is not affected by the nitrotyrosination of the peptide in H-2K^b^/NY-gp34 when compared to H-2K^b^/gp34.

In conclusion, substitution of the tyrosine residue at position 3 of the peptide gp34 to a nitrotyrosine exclusively affects the conformation of the side-chain of the H-2K^b^ residue E152. The specificity of the T cell hybridoma 24H1 to H-2K^b^/NY-gp34 thus seems to be dependent on the conformation of the MHC region surrounding the side-chains of p3NY and the H-2K^b^ residue E152. It should be noted that previous studies have demonstrated the importance of this region for recognition by H-2K^b^-specific TCRs [58], [59]. One may thus speculate that nitrotyrosination of the immunodominant peptide which alters considerably T cell recognition may be advantageous for the virus in order to escape immune recognition.

### Nitrotyrosination of gp34 strains the overall conformation of the H-2K^b^ complex and significantly decreases its stability

**Figure 4:**
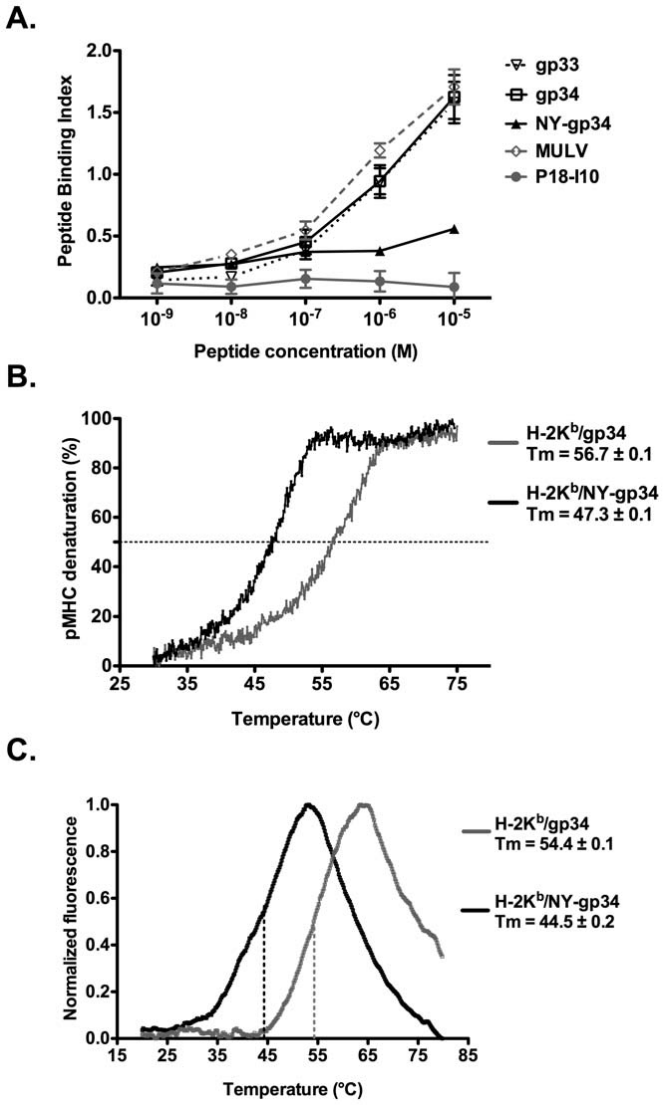
Nitrotyrosination of gp34 significantly decreases both its binding affinity and stabilization capacity of the H-2K^b^ complex. **A.** The binding affinity of NY-gp34to H-2K^b^on the surface of TAP-deficient RMA-S cells was significantly reduced when compared to gp33 and gp34. Cells were incubated with the indicated concentrations of each peptide at 26°C for 12 hours in 5% CO_2_. Cells were collected and stained with the H-2K^b^-specific antibody AF6-88.5. The mean fluorescence intensities (MFI) observed for each timepoint were normalized as described in the peptide binding affinity assays outlined above. The H-2D^d^ and H-2D^b^-restricted peptides P18-I10 and MulV were used as negative and positive controls, respectively. The peptide binding assays were repeated three times. **B.** The capacity of NY-gp34 to stabilize soluble H-2K^b^ complexes is significantly reduced when compared to gp34. MHC complex melting temperatures (T_m_) were measured using circular dichroism. T_m_ values were derived from normalized thermal denaturation curves for the MHC complexes H-2K^b^/gp34 (in grey) and H-2K^b^/NY-gp34 (in black) as the temperature corresponding to 50% denaturation (dashed line). The presented denaturation curves are an average from at least two independent measurements for each MHC complex. **C.** The denaturation curves of the two MHC complexes, using the differential scanning fluorimetry approach (thermofluor), are consistent with the two-state model of thermal unfolding. The melting temperatures, defined as the inflection points of the escalating parts of the curves, the curves of H-2K^b^/gp34 and H-2K^b^/NY-gp34 are in grey and black, respectively.

Two additional interactions are formed between peptide residue p3NY and the heavy chain residues Q114 and Y116 in the H-2K^b^/NY-gp34 peptide when compared to H-2K^b^/gp34 (Figures 3B and 3C), suggesting that nitrotyrosination of the peptide may result in higher stabilization capacity of H-2K^b^ complexes by NY-gp34 when compared to gp34. However, binding affinity assays using TAP-deficient cells demonstrated that while both gp33 and gp34 displayed similar binding affinities to H-2K^b^, nitrotyrosination of peptide residue 3 significantly reduced binding affinity of NY-gp34 to H-2K^b^ (Figure 4A).

Furthermore, the capacity of each peptide to stabilize H-2K^b^ complexes was also investigated using circular dichroism (CD) by assessing the thermostability of soluble H-2K^b^ molecules in complex with gp34 or NY-gp34. Melting temperatures (T_m_) were derived from changes in ellipticity at 218 nm, corresponding to loss of secondary structure during denaturation. Our analysis demonstrates that the T_m_ value for H-2K^b^/NY-gp34 (47.3°C) was nearly 10°C lower than for H-2K^b^/gp34 (56.7°C), reflecting the significantly reduced capacity of NY-gp34 to stabilize H-2K^b^ complexes (Figure 4B). Similar very significant shifts in thermal stability were also obtained for the two MHC class I complexes upon analyzing their stability with differential scanning fluorimetry (DSF, thermofluor) [60]. The melting temperatures T_m_ corresponding to the mid-point of the sharp transition from folded to unfolded state were 54.4±0.1 and 44.5±0.2 for H-2K^b^/gp34 and H-2K^b^/NY-gp34 (Figure 4C). These significant differences in stabilization capacity were at first surprising when considering that additional hydrogen bonds and long ionic interactions are formed between NY-gp34 and H-2K^b^ when compared to H-2K^b^/gp34 (Figure 3C). However, further comparative analysis of the two crystal structures provided an explanation to these unexpected results.

In H-2K^b^/NY-gp34, the side-chain of the nitrotyrosine p3NY projects within the D-pocket of H-2K^b^, resulting in structural over-packing (Figures 3A and 3C) and a ‘strained’ overall conformation of the MHC complex. Although this has not, to our knowledge, yet been reported for MHC class I molecules, strained conformations are relatively common and have been described for several proteins including e.g. native serine protease inhibitors [61] and spectrin SH3 core-domains [62]. Induction of strained conformations is also important within several processes including catalysis in thymidylate synthase [63] and nuclear transport of exportins [64]. Proteins with strained ‘spring-loaded’ conformations are usually intrinsically unstable. For example, the release of the course of the strained conformation in circularly permutated barnases resulted in a 20°C shift in melting temperature [65] or in a 25-fold increase in stability for mutated serpins [66]. In the crystal structure of the H-2K^b^/NY-gp34 complex, introduction of the side chain of p3NY resulted in close contacts between the nitrotyrosinated peptide and several H-2K^b^ residues, forcing them away from the relaxed low-energy conformation observed in H-2K^b^/gp34 in order to accommodate the side chain of p3NY, thus resulting in unfavorable strained conformations. Besides straining the backbone of the heavy chain residues 124–126 and 156–157, the side chain of p3NY also incurred strained higher internal energy conformations for the side-chains of residues V97, I98 and Q114, when compared to H-2K^b^/gp34.

In conclusion, the potential gain in enthalpy resulting from the formation of two additional interactions between p3NY and the H-2K^b^ residues Q114 and Y116 may be outweighed by the loss of a hydrogen bond interaction formed with the side chain of E152 combined with the free energy cost resulting from the strained conformations of several MHC residues. Our results indicate that formation of additional interactions between peptides and MHC residues does not always necessarily increase the overall stabilization of MHC complexes. Furthermore, alteration of MHC complex stabilization through nitrotyrosination of the presented epitope may represent an additional alternative strategy used by the virus to prevent adequate presentation of the peptide at the surface of the infected cell.

### Nitrotyrosination of p4Y in H-2D^b^/NY-gp33 directly affects recognition by H-2D^b^/gp33-specific TCRs

**Figure 5:**
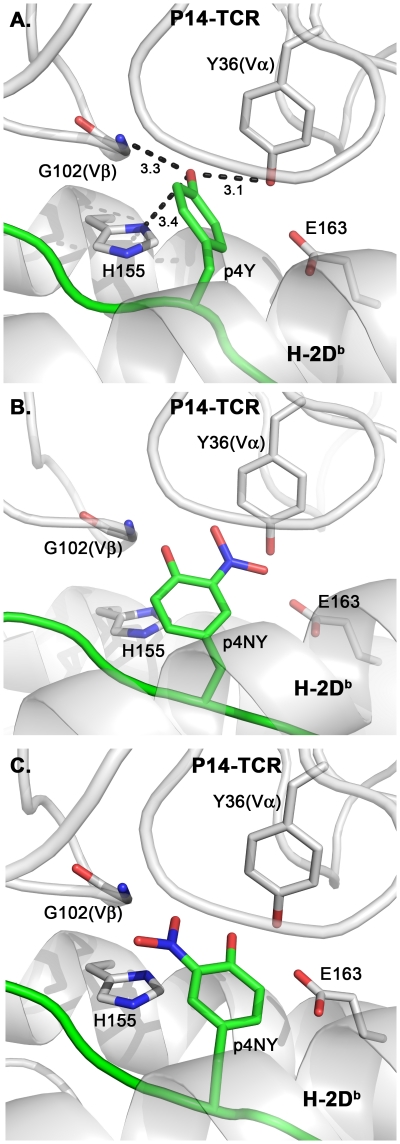
Nitrotyrosination of p4Y in H-2D^b^/NY-gp33 directly affects recognition by H-2D^b^/gp33-specific TCR. Nitrotyrosination of the main TCR-interacting peptide residue p4Y will affect the structural conformation of both TCR interacting residues on H-2D^b^ and of the TCR P14. The peptide binding cleft of H-2D^b^ and the TCR, both colored in white, are annotated. Hydrogen bond interactions appear as dotted lines. **A.** In H-2D^b^/gp33, the side chain of p4Y protrudes out of the H-2D^b^peptide-binding cleft, positioning itself perfectly in the hot spot of the p14 TCR composed of the CDR3 loops from both Vα and Vβ. It forms three hydrogen bonds, two of them directly with Y36(Vα) and G102(Vβ) on the TCR P14. The last hydrogen bond is formed with the side chain of the H-2D^b^ histidine residue H155, linking this domain of the heavy chain to the TCR. **B.** The side chain of the nitrotyrosinated p4-NY can not be accommodated within the hot-spot of P14, resulting in sterical clashes with the side chain of the TCR residue Y36(Vα). Furthermore, the negatively charged side chain of the H-2D^b^ residue E163, important for TCR recognition, would also be repelled by the introduced negatively charged nitrotyrosination. **C.** Similarly, the other rotamer of the nitrotyrosinated p4-NY would result in sterical clashes with both G102(Vβ)the side chain of H155, abolishing all formed hydrogen bond interactions.

The previously determined crystal structures of H-2D^b^ and H-2K^b^ in complex with gp33 demonstrated that the peptide binds in two diametrically opposed manners to the two MHC class I molecules [50]. Thus in contrast to H-2K^b^/gp33 and H-2K^b^/gp34, in which the side-chain of the tyrosine residue acts as a secondary anchor position, the side-chain of p4Y in H-2D^b^/gp33 protrudes out of the peptide-binding cleft, playing a key role in TCR recognition [41], [43], [50]. Indeed, mutation of p4Y to a phenylalanine (Y4F) reduced the affinity of the H-2D^b^/gp33-specific TCR P14 by 100-fold [51], [67]. A molecular model of P14 in complex with the crystal structure of H-2D^b^/gp33 indicated that the side-chain of the protruding p4Y is perfectly positioned in the hotspot of the TCR composed of the CDR3 loops from both the Vα and Vβ domains. It forms three hydrogen bonds with the TCR residues Y36(Vα) and G102(Vβ), as well as with the side chain of the H-2D^b^ histidine residue H155, linking this domain of the heavy chain to the TCR (Figure 5A). In contrast, insertion of the negatively charged NO_2_ group in p4NY would result in deleterious sterical clashes with Y36(Vα) or G102(Vβ) (Figures 5B and 5C). The side chain of one of the possible p4NY rotamers would also repulse the negatively charged side-chain of the MHC residue E163, important for TCR recognition (Figure 5B).

In conclusion, nitrotyrosination of p4Y in H-2D^b^/NY-gp33 is clearly deleterious for recognition by P14 as previously demonstrated [37]. Thus, one may here speculate again about the possibility that nitrotyrosination of the H-2D^b^-restricted peptide gp33 may represent an important strategy used by LCMV in order to avoid CD8^+^ T cell recognition and escape immune recognition. Furthermore, and as for H-2K^b^/NY-gp34, our structural analysis also strongly indicates that NY-gp33 may act as an H-2D^b^-restricted neo-epitope with the capacity to break tolerance, selecting and inducing inadequate T cell responses that may result in autoreactivity.

### Concluding remarks

Our study provides a structural platform underlying the effects of peptide nitrotyrosination on initiation of TCR responses. Such PTMs can directly affect TCR recognition by modifying the properties of key TCR-interacting residues on the presented peptide or by altering the conformations of other MHC residues that are of importance for TCR recognition. Nitrotyrosination can also indirectly affect TCR recognition by severely destabilizing the MHC complex. Although additional interactions were formed between the PTM peptide and H-2K^b^, the conformation of a large amount of surrounding residues was strained, significantly reducing the overall stability of the MHC complex. We have previously demonstrated that subtle modifications in MHC-restricted peptides can result in significant alterations of MHC stabilization [51], a phenomenon that should be taken into account and measured upon trying to design altered peptide ligands for modulation of TCR response [49], [68]. Several other amino acid modifications caused by oxidative stress could result in similar effects on TCR recognition [69], [70]. In conclusion, our structural study indicates that the impact of post-translational modifications could be dual, possibly allowing for viral immune escape from TCR recognition but also potentially inducing the expansion of subset of T cells that could induce autoreactivity. Likewise, nitrotyrosination of self-peptides during periods of inflammation or oxidative stress could lead to the formation of neo-epitopes that, through the same mechanisms described herein for gp33/34, could escape the constraints of central tolerance. The impact of these modified *de-novo* MHC complexes on the initiation of unwanted T cell responses that may result in autoreactivity remains to be studied.
